# Dyslexia treatment studies: A systematic review and suggestions on testing treatment efficacy with small effects and small samples

**DOI:** 10.3758/s13428-021-01549-x

**Published:** 2021-03-10

**Authors:** Enrico Toffalini, David Giofrè, Massimiliano Pastore, Barbara Carretti, Federica Fraccadori, Denes Szűcs

**Affiliations:** 1 Department of General Psychology, University of Padua, Via Venezia, 8, 35131 Padua, PD Italy; 2 DISFOR, University of Genoa, Genova, Italy; 3 Department of Developmental Psychology and Socialisation, University of Padua, Padova, Italy; 4 Department of Psychology, Centre for Neuroscience in Education, University of Cambridge, Cambridge, UK

**Keywords:** Dyslexia, Treatment efficacy, Design analysis, Meta-analysis, Mixed-effects modelling

## Abstract

Poor response to treatment is a defining characteristic of reading disorder. In the present systematic review and meta-analysis, we found that the overall average effect size for treatment efficacy was modest, with a mean standardized difference of 0.38. Small true effects, combined with the difficulty to recruit large samples, seriously challenge researchers planning to test treatment efficacy in dyslexia and potentially in other learning disorders. Nonetheless, most published studies claim effectiveness, generally based on liberal use of multiple testing. This inflates the risk that most statistically significant results are associated with overestimated effect sizes. To enhance power, we propose the strategic use of repeated measurements with mixed-effects modelling. This novel approach would enable us to estimate both individual parameters and population-level effects more reliably. We suggest assessing a reading outcome not once, but three times, at pre-treatment and three times at post-treatment. Such design would require only modest additional efforts compared to current practices. Based on this, we performed ad hoc a priori design analyses via simulation studies. Results showed that using the novel design may allow one to reach adequate power even with low sample sizes of 30–40 participants (i.e., 15–20 participants per group) for a typical effect size of *d* = 0.38. Nonetheless, more conservative assumptions are warranted for various reasons, including a high risk of publication bias in the extant literature. Our considerations can be extended to intervention studies of other types of neurodevelopmental disorders.

**Supplementary Information:**

The online version contains supplementary material available at 10.3758/s13428-021-01549-x.

## Introduction

### The issue of low power when assessing treatment efficacy in dyslexia

Poor response to treatment is a defining feature of specific learning disorders (American Psychiatric Association [APA], 2013). Therefore, even effective remediation programs may deliver only small improvements. For example, a recent meta-analysis of randomized controlled trials on dyslexia showed that all treatment approaches result in modest effect sizes (Galuschka, Ise, Krick, & Schulte-Korne, 2014). The estimated effect size (i.e., treatment-control standardized difference at post-test, corrected for the pre-test scores) for the most frequently used treatment approach as well as the only one for which a statistically significant meta-analytic estimate emerged, phonics instruction, was Hedge’s *g* = 0.32. This effect size can be considered relatively small (Cohen, 1988). In addition, this was further deflated to *g* = 0.20 after controlling for publication bias. The estimated effect sizes for the other treatment approaches ranged between 0.13 and 0.39, with a mean of 0.27. Therefore, large reported post-treatment improvements in children with dyslexia should probably be interpreted with caution. Large effect sizes may be due to noisy estimates (e.g., large standard error due to small sample size, unreliable reading measures) or inappropriate diagnostic procedures (e.g., children’s low reading performance being only transient and not due to dyslexia).

The difficulty in recruiting large enough samples, combined with a small true effect size, is often regarded as the major cause of low statistical power. The latter not only makes it difficult to distinguish true results from false positive results, but also inflates the risk of overestimating effect sizes. This risk can be defined a priori as the “exaggeration ratio” that indicates how much an effect size will be overestimated on the average in comparison with a plausible true effect size given that statistical significance is reached (e.g., Gelman & Carlin, 2014; see also Altoè et al., 2020). Unfortunately, researchers who test treatment efficacy in learning disorders frequently encounter this problem. In this field, recruiting large samples is difficult for different reasons. First, children with learning disorders represent only a subset of the general population. While this subset is epidemiologically relevant, it is small in absolute terms, totalling no more than 5–10% of all children (cf. DSM-5; APA, 2013). Second, studies on treatment efficacy require considerable compliance from children and their families, and several hours of their time, making it even more difficult to perform treatment studies with large samples.

Calculating power—and the exaggeration ratio—in this field may not be easy. It depends on several factors, including what inferential analysis is performed, pretest–posttest reading score correlation, and how the effect size is calculated. Concerning inferential analysis, the best choice is perhaps the use of linear models/ANCOVA on post-treatment scores, testing the effect of group and covarying the pre-treatment scores (e.g., Gelman, Hill, & Vehtari, 2020; Van Breukelen, 2006). Note that covarying pre-treatment scores here serve to increase power by controlling for the individual baselines, not to correct for initial group differences. Other methods such as testing the group by time interaction or comparing gain scores are also appropriate and lead to unbiased estimates, but they may have slightly less power (e.g., Dimitrov & Rumrill, 2003; Van Breukelen, 2006). The pretest–posttest scores correlation is importantly related to the reliability of the outcome reading measure that greatly affects power, as will be discussed in detail later.

The calculation of effect size is not trivial, and different formulae have been proposed. Reading scores, the skill targeted by intervention in most reading treatments, are quantitative continuous measures (e.g., reading time/speed, error rates). Thus, treatment efficacy can be expressed as a standardized difference between pre- and post-intervention scores. Morris (2008) suggests calculating the pre-to-post change in the treated group minus the pre-to-post change in the control group, all divided by the pooled standard deviation calculated from pre-test scores.

Most published randomized controlled trials investigating treatment efficacy in dyslexia are seriously underpowered under plausible assumptions. Reviewing the 22 published randomized controlled trials in the meta-analysis by Galuschka et al. (2014), we found 32 and 20 as the mean and median number of participants per group, respectively. However, assuming a real effect size of *d* = 0.27 (i.e., the unweighted average effect size across all treatment approaches calculated from Galuschka et al., 2014), a pretest–posttest correlation between reading scores of *r* = .80 (which may reasonably reflect the reliability of a reading measure used within a special population, see below), setting a conventional critical α = .05, and running the ANCOVA as suggested in the previous paragraph, then about 79 participants per group (i.e., minimum sample size of 158) are needed to reach a statistical power of 80% and to limit the exaggeration ratio to about 1.10. This and the following calculations were obtained via simulation using the R software (R Core Team, 2020), with 10,000 iterations. Further details on our simulations will be provided below in Study 2. The R code has been made publicly available (see the Open Practice Statement section).

With only 20 participants per group (i.e., the median number of participants per group in the studies reviewed by Galuschka et al., 2014), the power is only 28%, and the exaggeration ratio is 1.83. This means that under the assumptions outlined above, an effect size—calculated with the formula suggested by Morris (2008)—associated with statistical significance would be on average nearly twice as large as the true effect size. Using methods less powerful than linear models/ANCOVA with pre-treatment scores as covariate may lead to even larger overestimations of effects. For example, simply comparing post-treatment scores using ANOVA/*t* test (and ignoring pre-treatment scores) leads to much worse results, with only 13% power and an exaggeration ratio of 3.01. In brief, randomized controlled trials in this field not only are unlikely to reach statistical significance (even if the treatment is effective), but are also at risk of overestimating effect sizes.

Despite their lack of statistical power, most published studies with dyslexic participants claim that their treatments are effective. We reviewed the 22 randomized controlled trials included in Galuschka et al.’s (2014) meta-analysis. In their titles or abstracts, 17 studies claimed improved reading following the remediation program, four studies stated that results were inconsistent or that improvements occurred in cognitive aspects related to but different from reading, and one study concluded that the results failed to demonstrate any treatment-related improvement. This “optimism” is particularly worrisome considering the low statistical power of most of these studies.

One of the reasons behind the above confidence may be the liberal use of uncorrected multiple testing. We reviewed the 22 studies included in the meta-analysis by Galuschka et al. (2014), and we found that they tested four different outcomes of reading on average (median = 3). To quantify treatment efficacy, Galuschka et al. (2014) appropriately combined all reading outcomes within the same “group comparison” in each study (e.g., in a study with a treated vs a control group, all reading outcomes were combined into a single effect). Conversely, virtually all studies analysed reading outcomes separately. In these studies, the presence of an isolated significant comparison in one reading outcome, at one post-treatment time point, was typically used to support a claim about the efficacy of a specific treatment, even in the presence of statistically non-significant findings regarding other outcomes. This does not necessarily mean that most results are false positives, but that most claims may be supported by overestimated effects.

### Reliability is crucial, and repeated measurements can be the key to increase power

Collecting several reading outcomes raises the problem of correcting *p* values for multiple comparisons if the outcomes are analysed separately. However, combining effects from multiple measurements can be a key to obtain more precise estimates, and thus to increase power to some extent. In meta-analyses, effect sizes are combined across studies, providing more precise estimates than possible in single studies. Similarly, effects calculated from multiple outcomes could be combined even within a single study, providing more precise estimates than possible from a single measurement.

An additional way to increase precision is using highly reliable measures. The formulae by Morris (Morris, 2008) show how higher pretest–posttest correlation (*ρ*, a proxy of a measure stability/reliability) reduces the effect size variance. Unsurprisingly, higher pretest–posttest correlation increases power for ANCOVA on post-treatment scores (covarying by pre-treatment scores, as recommended by Van Breukelen, 2006, for pretest–posttest-controlled studies), or for any other analysis in which pre-treatment scores are included, such as testing the group × time interaction. This will be shown in Study 2 via simulation. In brief, stronger pretest–posttest correlation means smaller measurement error, thus more precise estimates of the effect and powerful statistical tests.

The test–retest correlation is generally very high for reading measures, but how can it be determined precisely? One may consider the test–retest correlation calculated in the normative population if a standardized test battery is used. However, this correlation may differ when calculated in special populations such as dyslexics. Specifically, the test–retest correlation of reading scores in dyslexia may be smaller than that of the normative population because of the shrinkage of the reading score range (dyslexic participant performs at the lower tail of the distribution). That a correlation decreases as the range of one variable reduces can be easily shown via simulation. For example, we can simulate two correlated sets of scores with *r* = .95 test–retest reliability from a hypothetical population. Simulating selecting a dyslexic subgroup we can then select the cases whose average scores are one standard deviation (SD) below the population mean. In this subgroup, the test–retest correlation drops to *r* = .77. Unsurprisingly, two recent randomized controlled trials, reviewed in Study 1, reported the test–retest correlation (Wang, 2017; Wang, Liu, & Xu, 2019), and specified that this measure was .94 according to the test battery, but only .81 and .78, respectively, when calculated in their own samples of children with dyslexia. In addition, Cirino et al. (2002) reported that the test–retest correlation among standard scores from major reading batteries ranged between .46 and .92, with a median of about .70, in a sample of 78 children with reading disability. This is below the reliability levels generally reported by standardized batteries (for example, 13 out of 40 studies that we reviewed in our meta-analysis in Study 1 reported test–retest correlations from the normative samples of the standardized batteries that they used, and the range of values was from .71 to .96).

Whatever the pretest–posttest correlation, statistical power can be enhanced by adding more information from repeated measurements. This could be done by assessing reading performance not once, but several times at pretest and several times at posttest. Dyslexia (and learning disorders in general) represents an ideal case because the ability of interest can be assessed using relatively simple tasks. In research, reading is generally assessed using word lists and non-word lists, measuring speed/time and/or accuracy. For such reading tasks, several parallel versions could be easily created ad hoc and administered. Parameters such as word frequency, length, and orthographic complexity are relatively easy to control, and should be equated across parallel versions. In addition, since reading tasks are similar to everyday life reading requests, it can be assumed that any practice effect induced by the repeated measurements remains negligible.

The idea investigated in this paper is to exploit the advantage of single-case experimental designs, where repeated measurements are used to estimate with precision the individual baseline (and change) of a measure (e.g., Krasny-Pacini & Evans, 2018), while at the same time keeping the focus on the population-level effect. In our Study 2, we hypothesized a scenario in which reading performance is repeatedly assessed at pre-treatment and at post-treatment for all children, showing how it leads to superior power as compared to the traditional design with any reading outcome measured once at pre-treatment and once at post-treatment.

### Aims of the present investigation

The present article includes two studies. Study 1 is a systematic review and meta-analysis that updates and extends the investigation by Galuschka et al. (2014) to provide a picture of the latest developments in this field. Results by Galuschka et al. (2014) were formalized and used as a set of Bayesian informed priors in our own analysis. In recent years, with several new treatments for children with reading problems, the number of published studies has grown considerably. In addition, in recent years several journals have improved their statistical standards and practices to some extent (Giofrè, Cumming, Fresc, Boedker, & Tressoldi, 2017). Study 2 is based on the results of our meta-analysis and examines how statistical power can be improved when studying treatment efficacy in dyslexia. We provide examples of different a priori design analyses conducted via ad hoc simulation, showing how power varies with sample size, test–retest correlation, and the study design. We show the advantage of a design based on collecting and combining several outcome measurements at any single time point at both pre-treatment and post-treatment over the traditional study design collecting a single measure per outcome at each time point. (Note that according to our suggestion, multiple closely spaced successive measurements would need to be taken at each spaced-out follow-up measurement point.)

## Study 1: A systematic review and meta-analysis of the recent findings

We conducted a systematic review and meta-analysis of studies assessing treatment efficacy published in the past eight years (January 2013 through June 2020). This time span was chosen to update the work of Galuschka et al. (2014) that included studies published until 2013. Our search and inclusion criteria were very similar to those of Galuschka et al. (2014). However, we identified more studies in our quantitative synthesis than did Galuschka et al. (2014), suggesting that there was a surge of interest in this field in the past few years. It should be noted that new treatment approaches emerged in these recent studies as compared to those reviewed by Galuschka et al. (2014), including methodologies inspired by new neuropsychological perspectives. Our primary aim was to provide an overview of the recently published literature with a focus on methodological and statistical practices.

### Method

#### Literature search and inclusion criteria

Articles published from 2013 through June 2020 were reviewed. The searched databases were APA PsycInfo, Scopus, and PubMed, as they were expected to include virtually all relevant literature. No further search of the grey literature was conducted, as we crucially aimed to review the characteristics of the published literature. The search keys used by Galuschka et al. (2014) in their search seemed appropriate, thus we used the same: (“dyslexia” OR “developmental reading disorder” OR “developmental dyslexia” OR “developmental reading disability” OR “reading disorder” OR “word blindness” OR “spelling disorder” OR “developmental spelling disorder” OR “specific spelling disorder”) AND (“treatment” OR “therapy” OR “therapeutics” OR “training” OR “remediation”); that is, at least one term in the first bracket combined with at least one term in the second bracket. The above terms were searched in title, abstract, and keywords.

We considered any study reporting quantitative data concerning treatment efficacy on individuals with dyslexia/reading disorder. The following criteria had to be met for inclusion: (a) the treatment approach can be of any type, but it must aim to improve reading performance as its ultimate goal; (b) the manuscript must be written in English or any other language understood by one of the authors (including Spanish, French, Italian, Portuguese, and Hungarian); (c) participants must either be clinically diagnosed with developmental dyslexia (or reading disability or reading disorder) or having a profile compatible with a reading disorder as reported by the authors; in the latter case, participants must have reading performance either below the 25th percentile or one standard deviation below the population mean as assessed using standardized tests in their mother tongue; (d) participants must be described as having normal intelligence or an IQ not below 70 (if reported); (e) any comorbidity or co-occurring condition is acceptable, but they must be compatible with dyslexia status (e.g., deafness, neurological conditions, intellectual disability in one or more participants, or low socioeconomic status as a predominant condition of the entire sample, are exclusion criteria); (f) the study must include at least one control group comprising individuals with dyslexia, who must either be untreated, waiting list, or active control (e.g., a placebo condition; no comparisons between alternative/competing treatments were considered); (g) group allocation must be randomized; however, studies which did not explicitly mention whether the allocation was randomized were still included and labelled as “unclear”; the analyses were later performed both with and without these studies; (h) participants’ reading ability must be assessed at least twice, including before (pre-test score) and after treatment (post-test score).

**Fig. 1:**
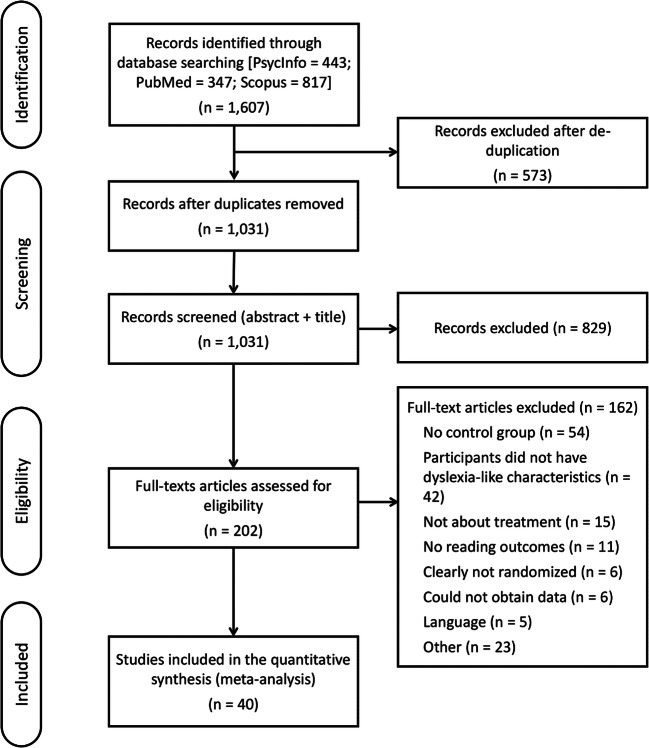
PRISMA flowchart

The PRISMA flow diagram summarizing the literature search and the selection process is reported in Fig. 1. The full-text eligibility assessment was conducted by two independent reviewers. The inter-judge agreement was good, Cohen’s k = .77. Disagreements were resolved via discussion with a third reviewer.

#### Coding of the studies

Two authors coded all studies and double-checked the entries. A different author further checked the final dataset. For each study, basic information including title, authors and year of publication were coded. The dataset included as many rows as effect sizes. An effect size was defined as standardized mean difference between reading scores in a treatment vs control group at the post-test (or follow-up), controlling for the pre-test scores. Effect sizes concerning follow-up assessments were coded if available but analysed separately. For most studies, more than one effect size could be calculated (e.g., because more than one reading outcome was used, or because there were multiple treated groups or control groups). Therefore, the dataset could include several observations per study.

For the calculation of the effect size, descriptive statistics of the reading scores (i.e., mean, standard deviation, and number of participants on which they were calculated) were coded for both treated and control group, at both pre- and post-test (or follow-up). Descriptive statistics were coded from tables or text where possible, or derived and approximated figures. Where no descriptive statistics were reported, we coded any alternative detail that allowed us to estimate the effect size and its variance (e.g., standardized model coefficients, effect sizes reported by the authors). If no such details were available, the authors were directly contacted.

In addition to the effect sizes, a series of sample and methodological details were coded. Sample details included the mean age of participants or age range, gender distribution, and mean IQ (where reported). Methodological details included type of reading outcome (characters [for Chinese participants], words, pseudowords, or text reading, lexical decision), treatment approach, duration of the intervention in weeks, duration of each session in minutes, and total number of sessions. The “subgroup comparison within study” was also coded. This is an identifier of the treated-vs-control group comparison. It served to distinguish among partially independent effects within the same study when more than one treated group or more than one control group were reported.

Treatment approaches were coded following the categories used by Galuschka et al. (2014), who followed the National Institute of Child Health and Human Development (2000) review, where possible. These include phonemic awareness instruction, phonics instruction, reading fluency training, reading comprehension training, auditory training, medical treatment, and coloured overlays. In our review, however, new approaches were introduced. These included: brain stimulation treatment (e.g., using transcranial direct current stimulation [tDCS] to stimulate reading-related brain areas); action video game trainings; visual/visual-attentional trainings with a neuropsychological approach; working memory training; modelling (a Bandura-inspired approach); reading acceleration program (a training aiming to improve eye movements, which is particularly used in non-alphabetic languages); vergence training; multisensory stimulation approaches; and mixed approaches (i.e., treatments that combine elements from several other approaches). Concerning statistical analyses, we coded how treatment efficacy on reading measures was tested, whether any correction for multiple comparisons was adopted, and whether power analysis was mentioned as the rationale for sample size. Two reviewers independently coded these aspects and subsequently resolved discrepancies through discussion.

#### Analytic strategy

The analytic strategy followed the recommendations by Borenstein, Hedges, Higgins, & Rothstein (2009). The R software (R Core Team, 2020) was used to perform all analyses. All plots were drawn with the “ggplot2” package (Wickham, 2016) of R. Meta-analytic estimates and meta-regressions were computed using random-effects models. A random-effects modelling approach was chosen because it allows us to better account for the expectably large heterogeneity in the effect size across studies (Borenstein et al., 2009). This approach assumes that the effect sizes are sampled from a normally distributed population of effects sizes, rather than all reflecting the same true effect size. To determine the heterogeneity across studies, we looked at the estimated standard deviation among the true effects across studies (known as τ; Borenstein et al., 2009).

Where the descriptive statistics were available, the effect size and its variance were calculated using the formula recommended by Morris (2008) for the “pretest–posttest-control group” designs. This consists of the mean post-test vs pre-test gain in the treatment group minus the post-test vs pre-test gain in the control group, divided by the pooled pre-test standard deviation. Where the descriptive statistics were not available (5% of the effects in our dataset), we used the effect sizes as reported by the authors (provided that they represented the difference in pre-post gain in the treated group minus the control group), but its variance was still calculated using the Morris (2008) formula. As the pretest–posttest correlation (*ρ*) was never reported, we assumed it to be .80 for the calculation of the effect variance. Any alternative value between .50 and .90 affected negligibly the point estimates, but they obviously affected the estimated precision of the effects, and thus heterogeneity (which was estimated higher for higher *ρ*). Effect sizes obtained from scores expressing performance negatively (e.g., reading times, errors) were sign-inverted for consistency.

Most studies reported more than one effect, and in many cases also more than one group comparison within study (i.e., comparison between a treated-vs-control pair of groups). These dependencies imply that effect sizes within the same study and within the same comparison within study provide partially redundant information, which must be accounted for. Therefore, we adopted a multilevel modelling approach, as implemented in the “brms” package of R (Bürkner, 2017). In our case, the multilevel structure was: Study > Group comparison within study > Effect size. In addition, but only to present the forest and funnel plot of the effects, and for simplicity in assessing the publication bias (see below), we combined the effect sizes within the same group comparison using the formulas for non-independent outcomes suggested by Borenstein et al. (2009; pp. 227–228). To compute the variance for a combined effect, the between-effect correlation was assumed to be *r* = .70. A sensitivity analysis showed that any alternative correlation between .30 and .90 had negligible effects on the point estimates.

Concerning moderators, we tested the age of participants (categorized as children [mean age below 18 years] or adults [mean age above 18 years]) and treatment intensity (in terms of total number of sessions and duration of treatment in weeks). They were tested via meta-regressions. Treatment approach, on the contrary, was not tested as a moderator, because there were very few studies for each single approach.

#### Bayesian estimation and definition of prior knowledge

A Bayesian approach to data analysis was adopted. We chose it because it enabled us to formalize and include prior information from the meta-analysis by Galuschka et al. (2014). For a full account of the advantages of a Bayesian approach, see Kruschke and Liddell (2018) and McElreath (2016). All meta-analytic models were fitted using the “brms” package of R (Bürkner, 2017), which uses the Markov chain Monte Carlo (MCMC) Bayesian estimation method implemented in the STAN programming language (Stan Development Team, 2018). All models presented below were run with four MCMC chains each with 5000 iterations, for a total of 10,000 post-burning effective iterations in each model. For any purpose of model comparison and statistical inference, the widely applicable information criterion (WAIC; Watanabe, 2010) was used (smaller values of WAIC indicate a better fitting model; McElreath, 2016). In examining any model coefficient, the mean value of its posterior distribution was taken as the point estimate, while its 95% Bayesian credible interval (BCI) was computed with the percentile method.

We defined prior knowledge from Galuschka et al. (2014). Prior distributions were defined only for the analysis concerning the pretest–posttest comparison, for the following parameters: the overall mean effect size, the heterogeneity across studies and across group comparisons within study, and for the estimated mean effect size of the specific treatment approaches that were considered both in our meta-analysis and in Galuschka et al. (2014). A “prior” indicates the probability distribution of an unknown parameter of interest (e.g., an effect size), before computing any analysis on the new data at hand.

To define the prior distributions for the overall mean effect size and its heterogeneity across studies, we reran a meta-analytic model including all 22 studies reviewed by Galuschka et al. (2014). For brevity, we have reported all details on the prior definition in the Supplemental material, Part 1. Here we report the prior distribution only for the mean effect size. It was set as Student’s *t* distributions with three degrees of freedom (a standard of the “brms” package of R) with M = .30, SD = .06. The central prior value for the τ coefficient of heterogeneity was .08, both at the study level and at the “group comparison within study” level (see details in Supplemental material, Part 1). Uninformed default priors were used for the moderators in the meta-regressions.

#### Assessment of publication bias

We used the precision-effect test and precision-effect estimate with standard errors (PET-PEESE), because it represents a less bad option among other conventional meta-analytic approaches (Stanley, 2017). However, assessing the publication bias with a limited number of studies, nearly all of them presenting small samples, and with predictably high heterogeneity, is difficult and any result must be taken with caution.

The PET-PEESE method consists of two conditional meta-regressions in which the standard error (PET) or variance (PEESE) are entered as moderators of the effect size. The regression coefficient is interpreted for evidence of the bias, whereas the model intercept can be interpreted as the bias-adjusted effect size estimate. The PET method is used first. It assumes a constant publication bias at all levels of precision, which is correct if the true effect is null. If the estimated effect size remains significant, however, the PEESE method is recommended, which assumes larger publication bias for less precise studies by using a quadratic model (Stanley, 2017).

**Fig. 2:**
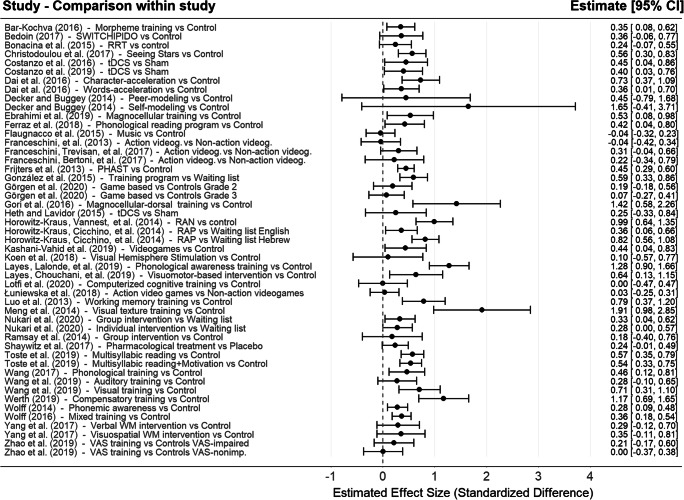
Descriptive forest plot of the effect sizes combined by group comparison within study. *Note*. Effect sizes within the same group comparison were combined assuming a between-effect correlation of *r* = .70 (alternative *r* values do not affect point estimates but affect their CIs)

**Fig. 3:**
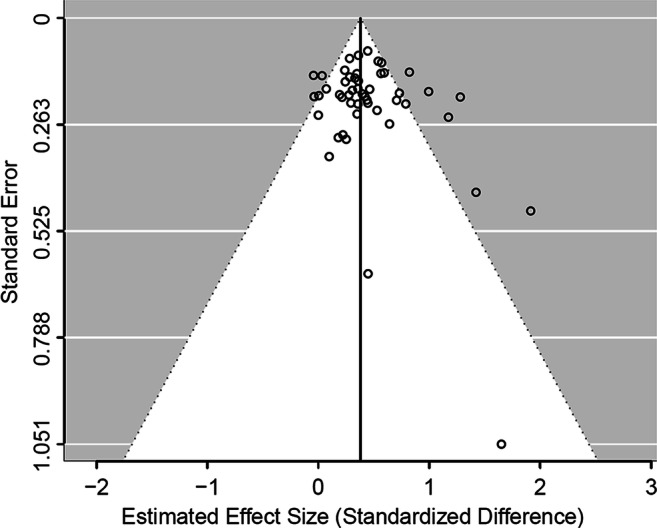
Funnel plot of the effect sizes combined by comparison within study

To avoid the further complication of multi-level modelling, the PET-PEESE meta-regressions were applied on the effect sizes combined by group comparison within study, and these were treated as independent. The formula for non-independent outcome suggested by Borenstein et al. (2009) was used to combine the effect sizes (see above in the Analytic Strategy section). Therefore, the PET-PEESE method was applied to the data shown in the forest (Fig. 2) and funnel (Fig. 3) plots. Furthermore, uninformed default priors were used for all model parameters when assessing the publication bias.

### Results

#### Overview and characteristics of the studies

A total of 40 studies met the criteria for being included in the quantitative analysis. Assignment of participants was explicitly randomized in 35 studies, and unclear in the remaining five studies. Thirty-one studies included one group comparison, and nine included two group comparisons. The latter subset included six studies presenting two treated groups compared against the same control group, and three studies presenting two treated groups each compared against a different, matched control group. All studies presented a pretest–posttest comparison; seven studies also reported a follow-up. Concerning the pretest–posttest comparisons, a total of 190 effect sizes were estimated. This meant an average of 4.8 outcomes per study and an average of 3.9 outcomes per group comparison within study.

An estimated total of 1862 participants were involved across the 40 studies, including 1103 treated and 759 control participants. The total number of groups was 90, including 49 treated and 43 control groups. The median and mean number of participants per group was 15 and 20.2 (treatment group: median = 15, mean = 22.5; control group: median = 15, mean = 17.7).

The estimated grand mean of the age of participants was 11.5 years (the range of estimated mean age of samples was 7.7–25.9 years). Thirty-six studies had participants in the developmental age (mean age between 7 and 14 years), and four studies had young adult participants (mean age between 22 and 26 years).

The median treatment session duration was 35 minutes, ranging between 15 and 450 minutes (including two studies with self-paced sessions). The median number of sessions per treatment was 18, ranging between 2 and 225 sessions. The median treatment duration was of 5 weeks, ranging between 1 and 30 weeks.

#### Meta-analytic estimates

The following analyses refer only to the pretest–posttest comparisons, except where indicated otherwise.

The overall meta-analytic estimate of the effect size, computed with multilevel modelling on 40 studies and a total of 49 group comparisons within study, was a medium standardized difference of *d* = 0.38 [95% BCI: 0.31, 0.46]. The estimated heterogeneity was substantial: across studies, *τ* = 0.12 [0.02, 0.24]; across group comparisons within study, *τ* = 0.17 [0.06, 0.27]. This means that, while the average effect size is estimated as 0.38, the true effect sizes across studies are estimated to range mostly between 0.14 and 0.62. The mean meta-analytic estimate obtained after excluding the five studies for which randomization was unclear remained virtually the same, *d* = 0.35 [0.28, 0.42].

The descriptive forest plot is shown in Fig. 2. A funnel plot is shown in Fig. 3.

The following treatment approaches were used in at least three different studies: phonemic awareness instruction, phonics instruction, mixed, brain stimulation, visual-attentional/neuropsychological, action video games, reading acceleration program, and working memory. Meta-analytic estimates calculated separately by these treatment approaches can be found in Supplemental material, Part 2. These estimates vary between .20 and .61; however, given the large heterogeneity of the effects, the very limited number of studies for each approach, and the resulting large BCIs, such estimates must be taken with caution. Each of the following remaining approaches was used in less than three studies: medical treatment, modelling, music training, reading fluency training, vergence.

There was no evidence in favour of the age class of the sample moderating the treatment efficacy, as shown in a multilevel meta-regression, *ΔWAIC* = +0.6. The estimated treatment efficacies confirmed that the effect was negligible: for children, *d* = 0.37 [0.30, 0.46]; for adults, *d* = 0.40 [0.13, 0.68]. There was no evidence in favour of a role of number of sessions, *ΔWAIC* = +0.8, |*B*| < 0.001, or a role of duration of treatment in weeks, *ΔWAIC* = -0.2, *B* = −0.004, as moderators of the treatment efficacy.

Finally, we analysed the pre-test vs follow-up comparisons which could be estimated from seven studies. Since all but one of these studies included only one group comparison, we entered only studies as the random effect. Uninformed default priors were used for all model parameters in this case. The meta-analytic estimate was *d* = 0.38 [0.23, 0.61]. Curiously, this is the same estimate as for the pretest–posttest comparison (but with larger uncertainty). The heterogeneity across studies was again large, *τ* = 0.14 [0.00, 0.46].

#### Publication bias

The PET-PEESE method suggested that there was publication bias. Concerning the overall effect size for the pretest–posttest comparisons, the PET model (including standard errors as a moderator of the effect size) fitted clearly better than the null model, *ΔWAIC* = −2.9; the moderator coefficient was *B* = 1.14 [0.03, 2.28]. The bias-adjusted estimated effect size was *d* = 0.22 [−0.01, 0.44]. As the 95% BCI included zero, this could be considered analogous to a “non-significant” estimate, and the PEESE model would conventionally be omitted. However, the interval of uncertainty for the bias-adjusted estimate is clearly not around zero, thus we do not believe that the null hypothesis can be accepted. Therefore, we still proceeded to fit the PEESE model. The latter had a better fit than the null model as well, *ΔWAIC* = −2.6 (moderator coefficient: *B* = 1.59 [0.08, 3.11]), but not better than the PET model, *ΔWAIC* = +0.3. The bias-adjusted effect size estimated by the PEESE model was *d* = 0.36 [0.26, 0.47], only slightly lower than the estimate from the null model, *d* = 0.42 [0.34, 0.51] (the latter estimate is larger than that presented in the previous section, but note that uninformed default priors were used here). In conclusion, evidence of a publication bias emerged, perhaps as large as to make it unclear whether the average true effect is non-null, but its precise extent remains uncertain.

Since the number of studies that included pretest–follow-up comparisons was very small, publication bias was not examined for this effect.

#### Details on analytical approaches

Most of the studies (i.e., 17 out of 40) assessed the treatment efficacy on reading outcomes via an ANOVA testing the group × time interaction, including three studies that also covaried for pre-treatment scores. ANOVA/linear models on post-treatment scores covarying pre-treatment scores were used by another seven studies. Analyses on pretest–posttest gains were conducted in only two studies. The remaining studies used a mix of methods, mostly including ANOVA/pairwise *t* tests/non-parametric tests to compare pretest–posttest scores differently by group or treated–control scores differently by time.

Nearly all studies used *p* value as the inferential criterion. Most of the studies (i.e., 33 out of 40) assessed multiple reading outcomes. Only 10 studies, however, adopted *p* value corrections (mostly using Bonferroni). Out of these, seven corrected for pairwise post hoc comparisons, and only three corrected for all comparisons (i.e., also for testing of multiple ANOVAs). Finally, out of the 33 studies that tested multiple reading outcomes, only two used multivariate ANOVA (MANOVA) to handle such multiplicity.

#### Considerations of statistical power and comparison with Galuschka et al. (2014)

It is apparent that the average number of participants per group has dropped over time. From the meta-analysis by Galuschka et al. (2014) to our present review, the median has dropped from 20 to 15, and mean has dropped from 32 to 20. This suggests that the previous meta-analysis failed to inform subsequent studies in terms of planning for adequate power. In fact, out of 40 studies that we reviewed, only three (Flaugnacco et al., 2015; González et al., 2015; Luniewska, Chyl, Debska, Kacprzak, & Plewko, 2018) mentioned and calculated power to justify their sample size. The three studies aimed to reach power levels between 80% and 99%, but all three seemed optimistic in their expectations. Specifically, one assumed a net (i.e., treatment minus control) effect size equivalent to *d* = 0.77 (without referring to the previous literature), one assumed an unspecified “medium to large effect size”, and another referred to previously reported effect sizes of medium magnitude (but it acknowledged that its sample size would be insufficient to detect a small effect).

Overall, statistical power was clearly insufficient in most studies. Based on our meta-analytic estimate of *d* = 0.38, assuming a pretest–posttest correlation of .80, testing statistical significance using ANCOVA covarying pre-treatment scores (Van Breukelen, 2006) and calculating the effect size as suggested by Morris (2008), the median study reviewed here (15 participants per group) had a power of 37%, with an exaggeration ratio of 1.58. Under these assumptions, a power of 80% would be exceeded with at least 43 participants per group (minimum sample size of 86). Unfortunately, only 5 out of 40 studies reviewed here had such a sample size or larger.

## Study 2: How to move on – simulating design analysis and using repeated measurements

**Fig. 4:**
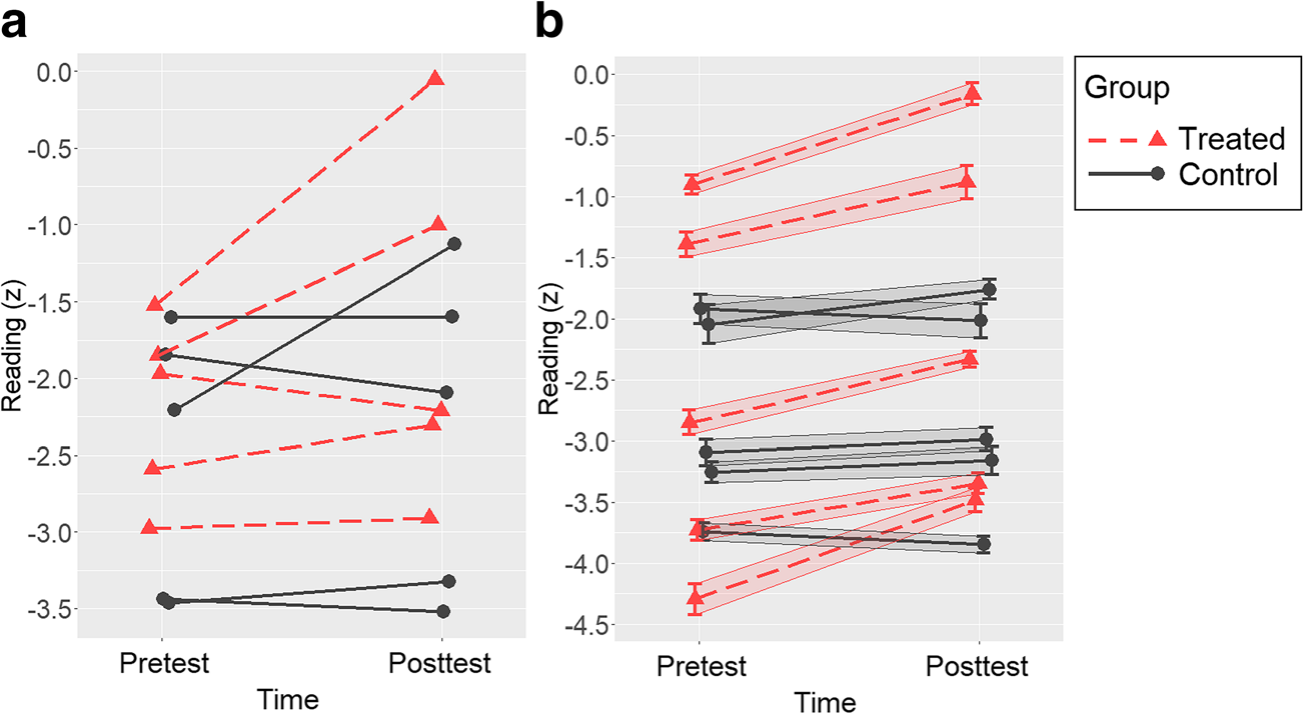
Illustration of the data that may emerge **a** from a traditional design measuring the individual reading performance only once at pre-test and at post-test and **b** from a design with repeated measurements and estimation with uncertainty of the individual parameters (error bars)

In Study 2 we conducted design analysis via simulation to examine how power (and the risk of overestimation) vary with measure reliability, use of repeated instead of one-off measurements, and inferential criterion (*p* value vs *Bayes factor*) when assessing treatment efficacy in dyslexia. As stressed in the Introduction, collecting repeated measurements at pre- and post-treatment may crucially increase reliability and thus power. Specifically, multiple measurements allow us to estimate both individual baseline and pretest–posttest variation with precision. This principle is illustrated in Fig. 4. Therefore, we conducted simulated design analysis also hypothesizing that reading performance would be assessed more than once at pre-treatment and at post-treatment.

### Method

All analyses described below were performed using R software and STAN, and the code is publicly available online.

#### Conducting design analysis via simulation

For simplicity, in our examples we assumed that reading scores would always be normally distributed (when measured both across different participants and within the same participant). Design analysis for a simple 2 (Group: treated vs control) × 2 (Time: pretest vs posttest) design, with one reading outcome measured once per time, requires assuming two most important parameters: the effect size and the pretest–posttest correlation. The pretest–posttest correlation was always set by generating paired arrays of correlated normally distributed scores for the pretest and posttest observations with the desired Pearson’s *r*. This correlation could be simulated in alternative ways, for example by determining the within- vs between-participant error variances. Nonetheless, we opted to directly generate correlated scores because a pretest–posttest correlation parameter is easier to formalize and to compare with values from the existing literature (e.g., based on the reliability scores of the test batteries). As the scores are sampled from a standard normal distribution, the standardized effect size was simply added to the post-treatment scores for the treated (and not for the control) group. However, one could easily simulate data on their real scale by linearly transforming all scores, or even generate non-normally distributed scores (e.g., correlated non-normally distributed scores can be simulated using the “semTools” package of R, Jorgensen, Pornprasertmanit, Schoemann, & Rosseel, 2020). Participants were assigned randomly to the treated or control group in all subsequent examples.

Response to treatment can be assumed to vary across individuals. To do so, the effect size can be sampled for each treated participant instead of being fixed. Assuming a normal distribution of effect sizes, this can be centred on a meta-analytic estimate (e.g., around 0.38 in our case), and have a plausible standard deviation (SD) that indicates how much the response to treatment may vary across participants. For example, sampling from *N*(0.38, 0.20) means that the large majority (about 95%) of treated participants would benefit from an effect that varies between about .00 and nearly .80 across individuals. This seemed plausible, so we used this distribution in all examples below. Nonetheless, we found that even an effect fixed to .38 for all treated participants virtually led to the same power levels. Simulating a treatment efficacy that varies across participants may be interesting if one plans to investigate individual differences in response to treatment, however, as we will comment in the Discussion section. Finally, an additional term could be added to the post-treatment scores of all participants to simulate a practice effect. Unless the practice effect is assumed to vary across participants, however, it will be practically negligible for both statistical power and the effect size calculation. In addition, practice effect in everyday-like reading tasks (e.g., reading a list of words) is likely negligible in children with dyslexia. Therefore, we did not consider it in the following examples.

Once a simulated dataset has been generated, the selected statistical analysis must be performed. In our case, we performed ANCOVA/linear models to test the effect of group on post-treatment scores, covarying pre-treatment scores (as suggested by Van Breukelen, 2006, but see also Gelman et al., 2020). However, one may use any alternative statistical methods of choice, for example testing group × time interaction, or using Bayesian estimating methods and inferential criteria. At this point, *p* value corrections should be applied to the simulated results if multiple reading outcomes are collected (in our simulated examples below, however, we assumed examining only one outcome). Concerning the estimation of the effect size, we used the formula suggested by Morris (2008).

To conduct design analysis, the entire process described so far must be repeated by several iterations (we opted for 10,000 iterations) for each of a series of alternative sample sizes. At each iteration, both the inferential criterion (e.g., *p* value from the ANCOVA) and the observed effect size (i.e., the effect size calculated on the simulated data) must be recorded. The entire process will end when the sample size leads to the desired level of power (e.g., more than 80% of iterations ending with *p* < .05 or with Bayes factor > 3) and/or to a desired level of the exaggeration ratio (e.g., the observed effect size associated with statistical significant being not larger than 10–15% more than the true effect size on average).

#### Use of repeated measurements

For the example concerning repeated measurements, we assumed that a reading outcome would be collected not once, but three times at pre-treatment and three times at post-treatment, which seems feasible in an experimental setting. Due to the structure of the data, ANCOVA/linear models covarying pre-treatment scores can no longer be performed in this case, unless scores are averaged by participant and by time (which we discourage for reasons explained below in the Discussion). Therefore, we assessed treatment efficacy testing the group × time interaction, which is still an appropriate choice (e.g., Dimitrov & Rumrill, 2003). Since repeated measurements were examined, we used mixed-effects models, fitted using the “lme4” package in R (Bates, Maechler, Bolker, & Walker, 2015). We entered group (treatment vs control) and time (pretest vs posttest) as the fixed effects of interest, participants as random effects (with random intercepts), and reading scores (without averaging) as the response variable. Random slopes were not fitted in this case, because the limited number of repeated measurements at each time point meant that they could not be estimated accurately. When we fit them in a separate simulation, however, the power was not affected to any visible extent. Nonetheless, fitting random slopes is recommended for larger numbers of repeated measurements (e.g., five or more).

The calculation of the empirical effect size in this case is not trivial. For simplicity, we applied the formula suggested by Morris (2008) despite multiple observations being collected for each participant at each time point. Alternatively, one could calculate the effect size on the data averaged by participant and by time. In the latter case, however, the observed effect size would somehow inflate, because averaging reduces the measurement error, thus decreasing the standard deviation.

#### Using Bayes factor instead of p value

None of the 40 studies that we reviewed in our meta-analysis used Bayesian methods. However, such methods are becoming increasingly popular in the social sciences. A fully Bayesian approach should encompass the definition of informed priors, the consideration of posterior distributions, and the discussion of the phenomenon at hand in probabilistic terms and in light of the prior expectations (e.g., Kruschke & Liddell, 2018; McElreath, 2016). Covering the complexity and the advantages of this approach, however, goes beyond the scope of the present article. Rather, we aimed to show how the design analysis for treatment efficacy in dyslexia is affected using a more simplified inferential procedure based on the Bayes factor (*BF*). Specifically, we used the popular “BayesFactor” package in R (Morey & Rouder, 2018) to fit Bayesian linear models and calculate the BF, with default settings.

Using BF as the inferential criterion does not affect the procedure for the simulation of the design analysis as described above, but it opens new inferential scenarios. Specifically, defining H_0_ as the null hypothesis (e.g., group × time interaction is null, or the effect of group on post-treatment scores is null) and H_1_ as the hypothesis that the treatment efficacy is non-zero, the BF can either support H_1_, support H_0_, or remain indecisive. Popular interpretive thresholds for the BF are the following: *BF* > 3 supports H1 with moderate (or stronger) evidence; *BF* < 1/3 supports H_0_ with moderate (or stronger) evidence; *BF* between 1/3 and 3 leaves one with inconclusive results or just anecdotal evidence (e.g., Raftery, 1995; Schönbrodt & Wagenmakers, 2018).

### Results

**Fig. 5:**
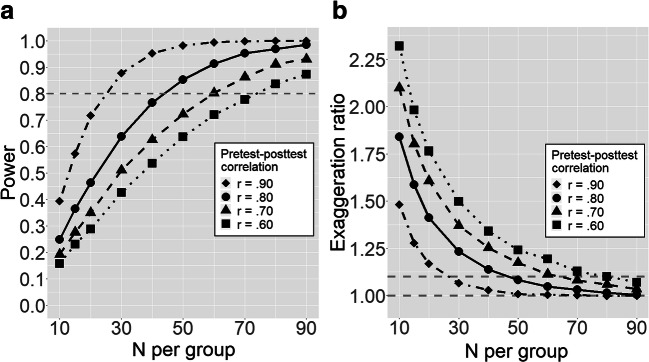
Design analysis showing **a** power and **b** exaggeration ratio for different numerosity of groups and pretest–posttest correlations for a treatment with a standardized effect size of *d* = 0.38, using a traditional experimental design (i.e., reading is measured once at pre-treatment and once at post-treatment for each participant). *Note*. The horizontal dashed lines represent in panel (**a**) the acceptable level of power = .80, and in panel (**b**) the perfect equivalence between estimated and true effect size (exaggeration ratio = 1.0) and the acceptable level of exaggeration ratio = 1.1

In a first analysis, we systematically examined how power and the exaggeration ratio vary with the pretest–posttest correlation (from .60 to .90) at different sample sizes (i.e., number of participants per group), using the traditional pretest–posttest-controlled design. We assumed a true effect of *d* = 0.38, which is in line with our meta-analytic results, and we used ANCOVA covarying pre-treatment scores, with a critical α = .05, for statistical inference. The results are shown in Fig. 5. As can be seen, higher pretest–posttest correlation of scores crucially enhances power.

**Fig. 6:**
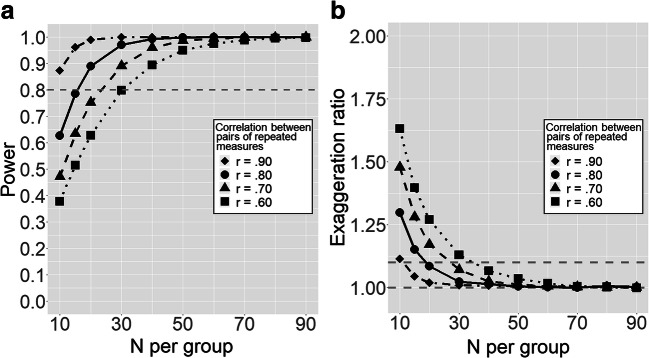
Design analysis showing **a** power and **b** exaggeration ratio for different numerosity of groups and different correlations between repeated measurements, for a treatment with a standardized effect size of *d* = 0.38, using a repeated-measurement experimental design with three distinct measures of reading at pre-treatment and three measures at post-treatment for each participant. *Note*. The horizontal dashed lines represent in panel (**a**) the acceptable level of power = .80, and in panel (**b**) the perfect equivalence between estimated and true effect size (exaggeration ratio = 1.0) and the acceptable level of exaggeration ratio = 1.1

In a second analysis, we focused on the use of repeated measurements with an outcome collected thrice at each time point. Again, we assumed an effect size of *d* = 0.38, and we varied the correlation between repeated measurements from .60 to .90. The latter parameter now describes the correlation between any pair of reading measures collected on the same participant (regardless of whether they were collected at pretest or posttest). Figure 6 shows the results. As can be seen, the power has clearly enhanced since the previous example. Specifically, for *r* = .80, Fig. 5 suggested that nearly 40 participants per group were needed for the power to exceed 80% (i.e., when only two groups are being compared, the total sample size needed is about 80), whereas Fig. 6 suggests that the same level of power could be reached with only about 15–20 participants per group using repeated measurements and mixed-effects models under the proposed scenario (i.e., with two groups, the sample size needed is 30–40 participants).

In a third analysis, we repeated the design analysis with both the traditional design and the suggested repeated measurements approach, but now using the *BF* instead of *p* value as the inferential criterion, and fitting models with the “BayesFactor” package in R. Once again, we set the effect size *d* = 0.38, and a repeated measures correlation of *r* = .80. As its default setting, a Cauchy distribution with scale = 0.50 was used as the prior for the standardized fixed effects (meaning that the prior was centred on zero, with half of its distribution beyond *d* < −0.50 or *d* > 0.50). Since it was unclear how mixed-effects linear models (and specifically the random effects) would be estimated using the “BayesFactor” package, we averaged scores by participant and by time in the case of repeated measurements, and we always used linear models on post-treatment scores, covarying pre-treatment scores (using frequentist methods as in the first two examples, we found that this alternative affected power negligibly).

**Fig. 7:**
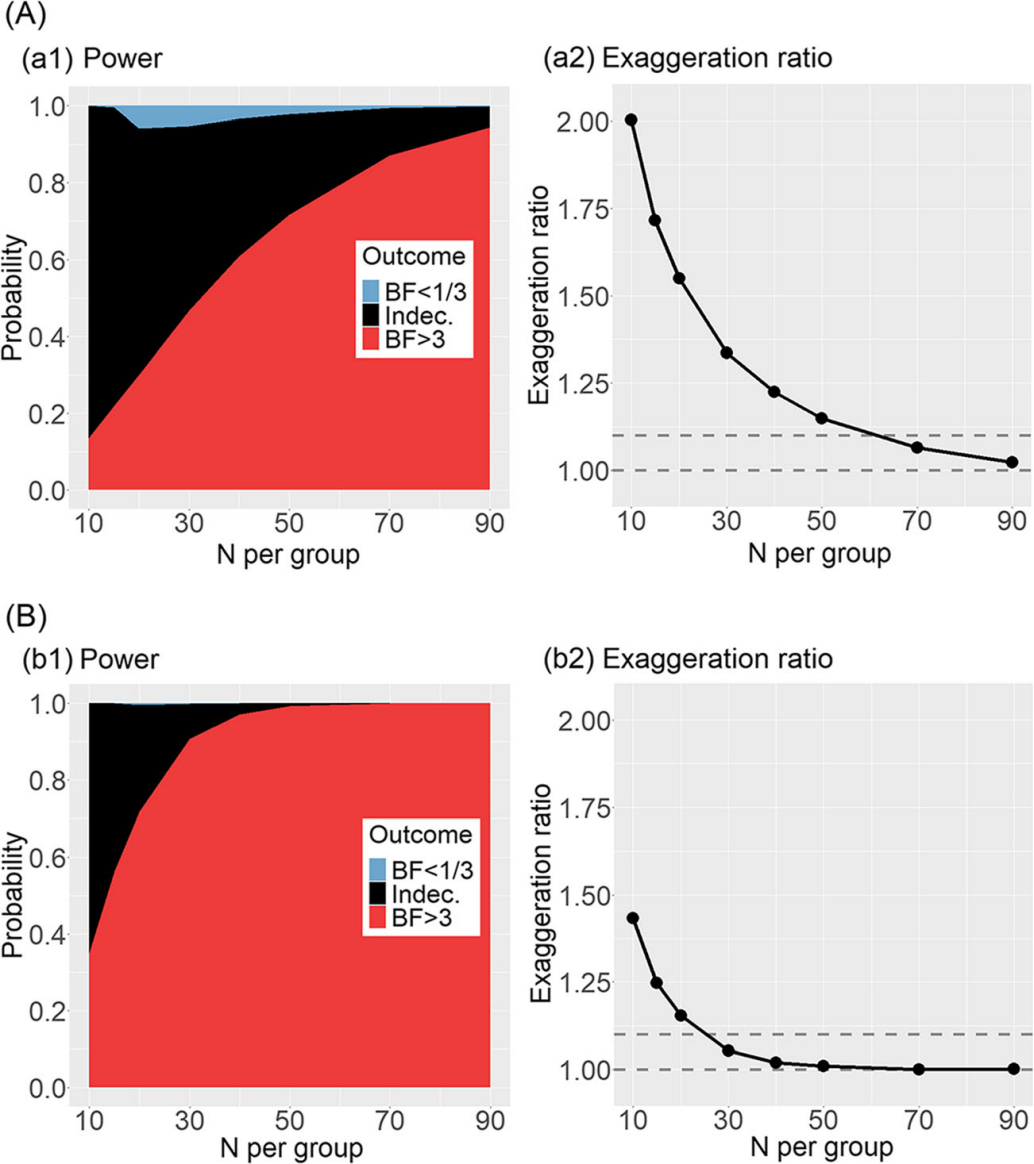
Design analysis for testing treatment efficacy using the Bayes factor (BF). True effect size is set as *d* = 0.38; correlation between repeated measurements is set as *r* = .80. Panel **A** refers to the classical design with a single measurement at pretest and posttest; panel **B** refers to an alternative design with three measurements of reading at pre-treatment and three measurements of reading at post-treatment for each participant

Figure 7 shows the results of the design analysis using *BF* for the traditional design (panel A) and for the repeated measures design that we proposed, with three reading measurements at pre-treatment and at post-treatment (panel B). Power was defined as the probability of supporting H_1_ with *BF* > 3. Unsurprisingly, Fig. 7 shows that the repeated measurement design was more powerful than the traditional design. Using the *BF*, however, did not increase power as compared to using frequentist methods (Figs. 5 and 6). This simply suggests that *BF* > 3 is roughly a stricter criterion than *p* < .05. An interpretive advantage of using the *BF* may be that, when H_1_ fails to be supported, it is possible to distinguish explicitly the case in which H_1_ can be rejected from the case in which the results remain indecisive. In Fig. 7, the “risk” of wrongly supporting H_0_ when the treatment is actually effective was around 5% for sample sizes up to 100 (i.e., up to 50 participants per group), but only in the classical design (panel A). In all other cases, regardless of the sample size, the predominant interpretive risk is that of remaining indecisive (BF between 1/3 and 3).

## Discussion

The first aim of this paper was to estimate the average effect size of treatments for dyslexia by performing an updated meta-analysis. The second aim was to provide recommendations on how to increase power when testing treatment efficacy in dyslexia, highlighting the importance of conducting a priori design analyses.

Concerning the first aim, the overall meta-analytic estimate for the pretest–posttest effect was *d* = 0.38, with a narrow 95% CI, which is encouraging. For the pretest–follow-up effect, the point estimate was the same. This is a larger effect size than the meta-analytic estimates of around 0.20–0.30 found by Galuschka et al. (2014), but it still qualifies as a modest effect. A notable difference between our results and those of the previous meta-analysis is the estimated precision of the effects. Our forest plot (Fig. 2) has error bars nearly 50% shorter, on average, than those presented by Galuschka et al. (2014), despite even smaller average sample sizes in our case. This is due to our calculation of the effect sizes using the formula proposed by Morris (2008), which incorporates the (assumed) pretest–posttest correlation in the estimated precision. As Galuschka et al. (2014) did not incorporate such information, their CIs are equivalent as assuming zero correlation between pretest and posttest scores that may not be a realistic assumption. Although this is unlikely to affect the meta-analytic point estimates of the effect size to a large degree, this clearly affects the estimated standard errors, and therefore confidence intervals, significance levels, and the estimated heterogeneity of the effect size across studies.

The 40 studies that we reviewed had a median participant number of 15 per group, corresponding to a median sample size of 30 for any single treatment-vs-control group comparison. This number is even smaller than the median across the 22 studies reviewed previously by Galuschka et al. (2014). It is in line with studies generally published in cognitive psychology and only slightly larger than the average sample size in neuroimaging studies (Szucs & Ioannidis, 2017, 2020). Nonetheless, we showed that with this median sample size, most studies in this field are seriously underpowered under any plausible assumptions. Furthermore, the PET-PEESE meta-regression method suggested that publication bias was likely. However, the relatively limited number of studies, the fact that nearly all of them had small sample sizes, and the substantial heterogeneity meant that the latter analysis may be unreliable (much like any other conventional approach of this kind; Stanley, 2017). The bias-adjusted estimate was deflated by nearly 50% (PET method) or less than 10% (PEESE method) vis-à-vis the non-adjusted estimate.

Despite their average statistical power being low, most studies that we reviewed in the present meta-analysis claimed that the proposed treatment was effective. Specifically, when we examined all titles and abstracts, we found that 33 out of 40 studies (83%) claimed that the treatment proposed was an effective remedy for participants with reading impairments. This may be because, as shown by our systematic review, most studies tested several outcomes without correcting *p* values for all multiple comparisons. This practice may result in many false positive findings even if statistically significant outcomes are found.

From a theoretical point of view, our review revealed that a variety of new treatment approaches have been used in recent years. A prominent one was the neuropsychologically inspired approach, including visual/visual-attentional trainings and action video games. Specifically, some studies focused on increasing visual-attentional skills that allegedly underpin reading ability, such as skills linked with the magnocellular pathway (e.g., Stein, 2018). Randomized controlled trials focusing on direct brain stimulation also emerged. Our results, however, suggested that these novel approaches were not more effective than the traditional ones on average (see Supplemental material, Part 2).

Concerning the second aim, we showed that under plausible assumptions, the sample size required to obtain a power of 80% is about double the median sample size in published randomized controlled trials. Nonetheless, it could be considerably reduced through a strategic use of repeated measurements. By plausible assumptions we meant a standardized effect size of *d* = 0.38, which corresponds to the overall mean estimate that resulted from our meta-analysis, and a pretest–posttest correlation of around *r* = .80. In fact, a more conservative effect size assumption (i.e., between 0.20 and 0.30) should be preferred by those who plan to assess treatment efficacy, both for caution if any novel approach is examined, and because our meta-analysis suggested the presence of a publication bias in the previous literature. Furthermore, in all our examples we assumed that only one reading outcome would be tested, thus without need to correct *p* value for multiple tests. Note that any *p* value correction, by requiring stricter inferential criteria, would further reduce power and increase the exaggeration ratio.

Given the importance of the test–retest/pretest–posttest correlation parameters, they should always be considered when testing treatment efficacy. Concerning the test–retest correlation, the actual reliability of standardized reading measures may be higher than .80 in normative populations. However, as explained in the Introduction, there are reasons to think that this value may not be as high as .80 in children with dyslexia. Calculating such parameter from one’s own experimental sample is an option, but since sample sizes are generally small, any estimate will probably be imprecise. It is worth noting that the classical test–retest correlation may reflect not only the stability in measuring the underlying construct of interest (i.e., reading ability), but also task-specific features. Considering the correlation among different parallel versions of a task is better than considering the test–retest correlation for the same version. An even better approach could be measuring the latent reading ability factor using a variety of different tasks. In this case, structural equation modelling, although typically requiring larger samples, may be the appropriate analytical approach. This is a venue for future research.

Concerning experimental design, we showed the advantage of collecting repeated measurements of individual reading scores at both pre-treatment and post-treatment times. This allows us to exploit the advantage of a single-case experimental design approach, in terms of having robust estimates of the individual parameters, while keeping the focus on the population-level effect. It is well known that increasing the number of observations might also lead to an increment of the statistical power and of the precision, even with the same number of participants (e.g., Maxwell, Delaney, & Kelley, 2018). Nonetheless, none of the 40 studies that we reviewed here adopted the design that we proposed, and only one (Wolff, 2014) did something similar by combining different reading outcomes in a latent factor using structural equation modelling (which typically requires large sample sizes, however).

We suggest that repeated measurements should be collected using different versions of a same task. In fact, while practice effects for everyday-like reading requests such as those posed by classical reading tasks (e.g., reading word lists) are likely negligible for children with dyslexia, we cannot exclude that it may become an issue when repeatedly presenting the exact same stimuli. Luckily, creating different versions of reading tasks is relatively easy because the material generally consists of simple verbal stimuli (controlled for a few important parameters such as word frequency, length, and orthographic complexity).

Concerning data analysis, we suggest using mixed-effects models, with participants as random effects, to exploit the information available with the recommended repeated measures design. The population-level effect (i.e., the average pretest–posttest gain) can be examined by considering the fixed-effects part of the model. However, the random-effects part can be of interest as well. Examining random slopes (which we did not do here for simplicity) represents an ideal approach for precisely estimating how individuals may respond differently to treatment. In fact, previous studies have suggested that response to treatment in dyslexia likely reflects individual differences (e.g., Aravena, Tijms, Snellings, & van der Molen, 2016; Zhao et al., 2019). Accurately investigating the variability in the random slopes, however, may require many repeated measurements of a reading outcome per time point, which is something that can be considered in a simulated ad hoc design analysis. An easier alternative to mixed-effects models could be fitting linear models on the scores averaged by participants and by time. However, this latter option loses information on intra-individual variability.

Using repeated measurements opens further questions and areas of investigation. It raises the issue of how reading performance varies intra-individually. How do reading measurements vary over time in the short- and medium-term? For example, do circadian oscillations affect reading performance, as they have been shown to affect other cognitive abilities (e.g., Hahn et al., 2012), especially in children with dyslexia? The ideal temporal spacing of repeated measurements within the same time point is a matter for future research.

Concerning the inferential criteria, we briefly examined how the use of a Bayesian criterion to quantify evidence may affect the design analysis. Using *BF* with default parameters did not help increasing power as compared to traditional frequentist methods. It may have the interpretive advantage of distinguishing between an uncertain outcome and a case in which H_0_ is supported by the *BF*. If the study is adequately powered for an effect size of interest, however, failing to reject H_0_ should imply rejecting H_1_ even using a frequentist approach. Furthermore, proper design analysis with *BF* should be conducted also under a null hypothesis scenario (e.g., Schönbrodt & Wagenmakers, 2018), to check whether H_0_ would be consistently supported—should it be true—under the chosen assumptions. In any case, if Bayesian inference is used, we recommend adopting a fully Bayesian approach, including an explicit formalization of the priors and the consideration of the posterior probability of the effect size (e.g., McElreath, 2016), rather than using a simplified criterion such as the *BF* calculated with the default parameters set by the software.

From a methodological point of view, we recommend adopting a simulation approach to design analysis. Although there are tools for the analytical calculation of power for a variety of statistical methods, simulation allows maximum flexibility. The latter is crucial because, as discussed, both power and the exaggeration ratio depend on several aspects, which may be difficult to control analytically. Via simulation, one can perform design analysis under several alternative ad hoc scenarios, for example assuming individual heterogeneity in response to treatment, varying the correlations among the outcome variables (or among the predictors in non-experimental settings), adopting alternative criteria for inference (e.g., *p* value vs *Bayes factor*), and considering not only power, but also other parameters such as the risk of overestimation (exaggeration ratio; Altoè et al., 2020; Gelman & Carlin, 2014) or the risk of supporting the wrong hypothesis (especially if Bayesian inference is used).

Finally, although we stressed the importance of a priori design analysis, we would like to warn the readers about its limitations. Specifically, our simulation analysis in Study 2 may have suggested that under ideal conditions (i.e., enough repeated measurements of the outcome at each time point combined with high measure reliability), high power may be reached even with very small samples. This may not always be the case, however. Data collected on small samples could still be unreliable for unforeseen reasons. First, as mentioned above, the time spacing between repeated measurements matters. Repetitions too close in time may lead to an extremely high correlation between measurements collected within the same time point (without increasing the correlation between pre-treatment and post-treatment). In this case, repeated measurements would be redundant, thus failing to enhance precision. The recommended design is most advantageous when such correlation is not too high (ideally, when it is as high as the correlation between pre-treatment and post-treatment scores), thus effectively serving to reduce the measurement error in the individual estimates of the underlying reading ability. Second, if response to treatment were largely heterogeneous across individuals, no result from any small sample could be generalized to the population, regardless of the precision of the estimates within the studied sample. For these reasons, we would not recommend planning to use sample sizes below 40 (i.e., below 20 participants per group), even under ideal conditions.

## Conclusions

Testing small samples is often unavoidable in studies on neurodevelopmental disorders, including randomized controlled trials assessing treatment efficacy in learning disorders. Unfortunately, plausible effect sizes in this field are also small. This is true not only because the real effect sizes in psychology are generally limited (Open Science Collaboration, 2015), but also because learning disorders are characterized by poor response to treatment by definition (DSM-5; APA, 2013). This combination of small samples and small true effects leads to low statistical power. The latter not only makes it difficult to distinguish true- from false-positive results (Szucs & Ioannidis, 2017), but also leads to an increased risk of overestimating the effect sizes (Gelman & Carlin, 2014), the so-called winner’s curse effect (Young, Ioannidis, & Al-Ubaydli, 2008; see also Button et al., 2013). This means that low-powered studies risk either reporting effects that are statistically significant but overestimated or reporting accurate effect size estimates that fail to reach statistical significance.

To enhance power, we suggest that researchers assessing treatment efficacy in dyslexia could exploit the advantages of estimating individual parameters with precision, like in the single-case experimental designs (e.g., Krasny-Pacini & Evans, 2018), but keeping the focus on the population-level effects. Using stable and reliable reading measures that ensure high pretest–posttest correlation is important. However, further benefit may come from collecting several measurements of reading performance both pre-treatment and at post-treatment for all participants. We showed that, under reasonable assumptions, even three distinct measurements at pretest and three measurements at posttest, analysed using mixed-effects models, may crucially increase power.

In conclusion, increasing power when testing treatment efficacy is challenging, but small effects may still be detected reliably, even with modest samples. We chose to focus on the treatment of dyslexia because of the increasingly large body of literature in this field and because reading ability is relatively easy to assess. However, all considerations presented here could be extended to other types of learning disorders (e.g., dyscalculia), or even to other neurodevelopmental disorders. In fact, outcomes different from reading may be more difficult or time-consuming to collect repeatedly at each time point. Many measures in the psychological research present even lower reliability than reading measures (e.g., mathematics or other related fields), however, meaning that the benefit of increasing precision by collecting several repeated observations, as suggested in the current report, might be even larger in areas outside the reading research. In any case, a priori design analysis, including the discussion and formalization of all expectations about the phenomenon at hand, is fundamental. For complex designs such as those described in this article, we suggested running ad hoc simulations to maximize flexibility. Finally, whatever a priori assumptions are made, all parameters formalized in the design analysis (e.g., pretest–posttest correlations) should later be checked against the empirical data. Should any large divergence emerge, the design analysis may need to be reconsidered and adjusted retrospectively.

## Supplementary Information

ESM 1 (DOCX 34 kb)
