# Supplementary material for: Dyslexia treatment studies: A systematic review and suggestions on testing treatment efficacy with small effects and small samples
Source: Behav Res Methods. 2021 Mar 10;53(5):1954–72. doi: 10.3758/s13428-021-01549-x (PMC8516770; doi:10.3758/s13428-021-01549-x)
Supplement: Supplementary file 1 — (DOCX 34 kb) [file 13428_2021_1549_MOESM1_ESM.docx]

**Dyslexia Treatment Studies: A Systematic Review and Suggestions on Testing Treatment Efficacy with Small Effects and Small Samples**

**Supplemental Materials**

**PART 1 – Details on the Definition of Prior Knowledge**

Prior distributions were defined for the average effect size concerning the pretest-posttest comparisons and, where possible, for its heterogeneity (τ parameter) across studies and across group comparison within study. For all parameters not specified below, uninformed default priors were used, as set by the STAN language. The prior distributions for the effect size *d* were modelled using Student’s t distributions with 3 degrees of freedom, which is a standard in the STAN language for both intercepts and beta parameters. Student’s t distribution is like a Normal distribution, but with heavier tails, thus allowing larger deviations from the mean. The *τ* parameters of heterogeneity, which represent standard deviations and must be defined on the positive domain, were modelled using Gamma distributions.

**Overall Effect Size and Its Heterogeneity**

The prior distributions for the overall mean effect size and its heterogeneity were defined by running a meta-analytic model including all 22 studies reviewed by Galuschka et al. (2014). The model was fitted with the “brms” package of R, using default uninformed priors. We had to do so because the authors never reported an overall meta-analytic effect size that included all studies simultaneously. Specifically, we coded the data reported by Galuschka et al. (2014) in their overview in Figure 2, and we run the model on it.

The estimated mean effect size was *d* = 0.30, with standard error (*SE*) = 0.06. These parameters were thus used as *M* and *SD*, respectively, for the Student’s t-distribution that defined the prior for the overall effect size in our meta-analysis.

Concerning the heterogeneity, the same model estimated *τ* = 0.08, *SE* = 0.05, for the variability at the study level, and *τ* = 0.08, *SE* = 0.06 for the variability at the “group comparison within study” level. Such estimates were used as the means and standard deviations of the prior distributions for the two levels of heterogeneity. Since the Gamma distribution was used, the *α* (*shape*) and *β* (*rate*) parameters were set to exactly reproduce the wanted means and standard deviations. Specifically, for the variability at the study level, *α* = 2.56, *β* = 32.00; for the variability at the “group comparison within study level”, *α* = 1.78, *β* = 22.22.

**Effect Size Divided by Treatment Approach**

Concerning the analyses separate by treatment approach, only “phonics instruction” and “phonemic awareness instruction”, among all approaches reviewed by Galuschka et al. (2014), were represented in at least three studies that we reviewed. For the mean effect size of these two treatment approaches, we directly used the estimates provided by Galuschka et al. (2014) in their Table 1 and elsewhere in their paper. The standard errors, which serve as the *SD* for our prior distribution, however, were not reported by the authors. Therefore, we had to compute them back from the 95% CI bounds.

For “phonemic awareness instruction”, we set an informed prior distribution with *M* = 0.28, *SD* = 0.27. For “phonics instruction”, we set an informed prior distribution with *M* = 0.20, *SD* = 0.08. The latter mean does not represent the raw estimate provided by Galuschka et al. (2014) for this treatment approach, but the estimate adjusted for the publication bias as reported by the authors. For all other treatment approaches that we investigated, there was no specific prior information in the meta-analysis by Galuschka et al. (2014). Therefore, we defined weakly informed prior distributions centred on the point estimate of the overall effect size (as defined in the previous section, i.e., *M* = 0.30), but with very large uncertainty, *SD* = 0.50. In this way, the prior distribution is centred on a plausible mean value, but its span of possible values ranges from well below zero to well above 1.00.

**PART 2 – Treatment Efficacy Divided by Treatment Approach**

Here we report estimated effect sizes for treatment efficacy divided by treatment approach. This was limited to the treatment approaches for which there were at least three studies each. Details on data analysis are reported in the manuscript. Prior distributions were defined in the previous section of this Supplemental materials document. Results are reported below in Table S1.

**Table S1**

*Estimated efficacy of treatment approaches on reading performance*

| **Treatment approach** | **N. of studies** | **N. of group comparisons** | **Median N per group** | **Prior distribution for the effect size**  **Student’s t (*df* = 3)** | **Estimated effect size** | **Std.**  **Err.** | **95% BCI** | | **τ^†^** |
| --- | --- | --- | --- | --- | --- | --- | --- | --- | --- |
|  |  |  |  |  |  |  | **LL** | **UL** |  |
| Phonemic awareness instruction | 3 | 3 | 20 | *M*=0.28, *SD*=0.27 | 0.58 | 0.15 | 0.29 | 0.87 | .12 |
| Phonics instruction | 7 | 8 | 23 | *M*=0.20, *SD*=0.08 | 0.44 | 0.07 | 0.27 | 0.57 | .09 |
| Mixed | 3 | 4 | 35 | *M*=0.30, *SD*=0.50^*^ | 0.25 | 0.09 | 0.07 | 0.42 | .08 |
| Brain stimulation | 3 | 3 | 11 | *M*=0.30, *SD*=0.50^*^ | 0.37 | 0.12 | 0.15 | 0.59 | .07 |
| Visual-attentional/Neuropsy. | 8 | 11 | 10 | *M*=0.30, *SD*=0.50^*^ | 0.35 | 0.07 | 0.22 | 0.51 | .10 |
| Action video game | 5 | 5 | 10 | *M*=0.30, *SD*=0.50^*^ | 0.20 | 0.11 | 0.01 | 0.43 | .10 |
| Reading acceleration program | 3 | 5 | 15 | *M*=0.30, *SD*=0.50^*^ | 0.61 | 0.11 | 0.38 | 0.83 | .08 |
| Working memory | 3 | 4 | 12 | *M*=0.30, *SD*=0.50^*^ | 0.36 | 0.13 | 0.10 | 0.61 | .09 |

*Note*. The analysis was limited to treatment approaches for which there were at least 3 studies.

^*^ Weakly informed prior centered on the overall mean effect size for plausibility.

^†^τ estimates the heterogeneity and it represents the estimated SD of the true effects across studies.

As for none of the treatment approaches there was a substantial number of studies (the maximum is k = 8), these results must be taken with much caution, particularly when considering the large heterogeneity (τ estimates), and the general risk of publication bias highlighted in the manuscript. The publication bias was not examined separately by treatment approach because the number of studies for any single approach was limited, making it difficult to evaluate this effect.

It is also worth to note that, in order to aggregate as many studies as possible, some specific categories of treatment approach presented in Table S1 were introduced by the authors of the current report and do not necessarily reflect the terminology used in the original studies.
